# A Comprehensive Data Gathering Network Architecture in Large-Scale Visual Sensor Networks

**DOI:** 10.1371/journal.pone.0226649

**Published:** 2020-01-07

**Authors:** Jing Zhang, Pei-Wei Tsai, Xingsi Xue, Xiucai Ye, Shunmiao Zhang

**Affiliations:** 1 School of Information Science and Engineering, Fujian University of Technology, and Fujian Provincial Key Laboratory of Big Data Mining and Applications, Fuzhou, China; 2 Department of Computer Science and Software Engineering, Swinburne University of Technology, Hawthorn, Australia; 3 Department of Computer Science, University of Tsukuba, Tsukuba, Japan; King Abdulaziz University, SAUDI ARABIA

## Abstract

The fundamental utility of the Large-Scale Visual Sensor Networks (LVSNs) is to monitor specified events and to transmit the detected information back to the sink for achieving the data aggregation purpose. However, the events of interest are usually not uniformly distributed but frequently detected in certain regions in real-world applications. It implies that when the events frequently picked up by the sensors in the same region, the transmission load of LVSNs is unbalanced and potentially cause the energy hole problem. To overcome this kind of problem for network lifetime, a Comprehensive Visual Data Gathering Network Architecture (CDNA), which is the first comparatively integrated architecture for LVSNs is designed in this paper. In CDNA, a novel *α*-hull based event location algorithm, which is oriented from the geometric model of *α*-hull, is designed for accurately and efficiently detect the location of the event. In addition, the Chi-Square distribution event-driven gradient deployment method is proposed to reduce the unbalanced energy consumption for alleviating energy hole problem. Moreover, an energy hole repairing method containing an efficient data gathering tree and a movement algorithm is proposed to ensure the efficiency of transmitting and solving the energy hole problem. Simulations are made for examining the performance of the proposed architecture. The simulation results indicate that the performance of CDNA is better than the previous algorithms in the realistic LVSN environment, such as the significant improvement of the network lifetime.

## Introduction

**Fig 1:**
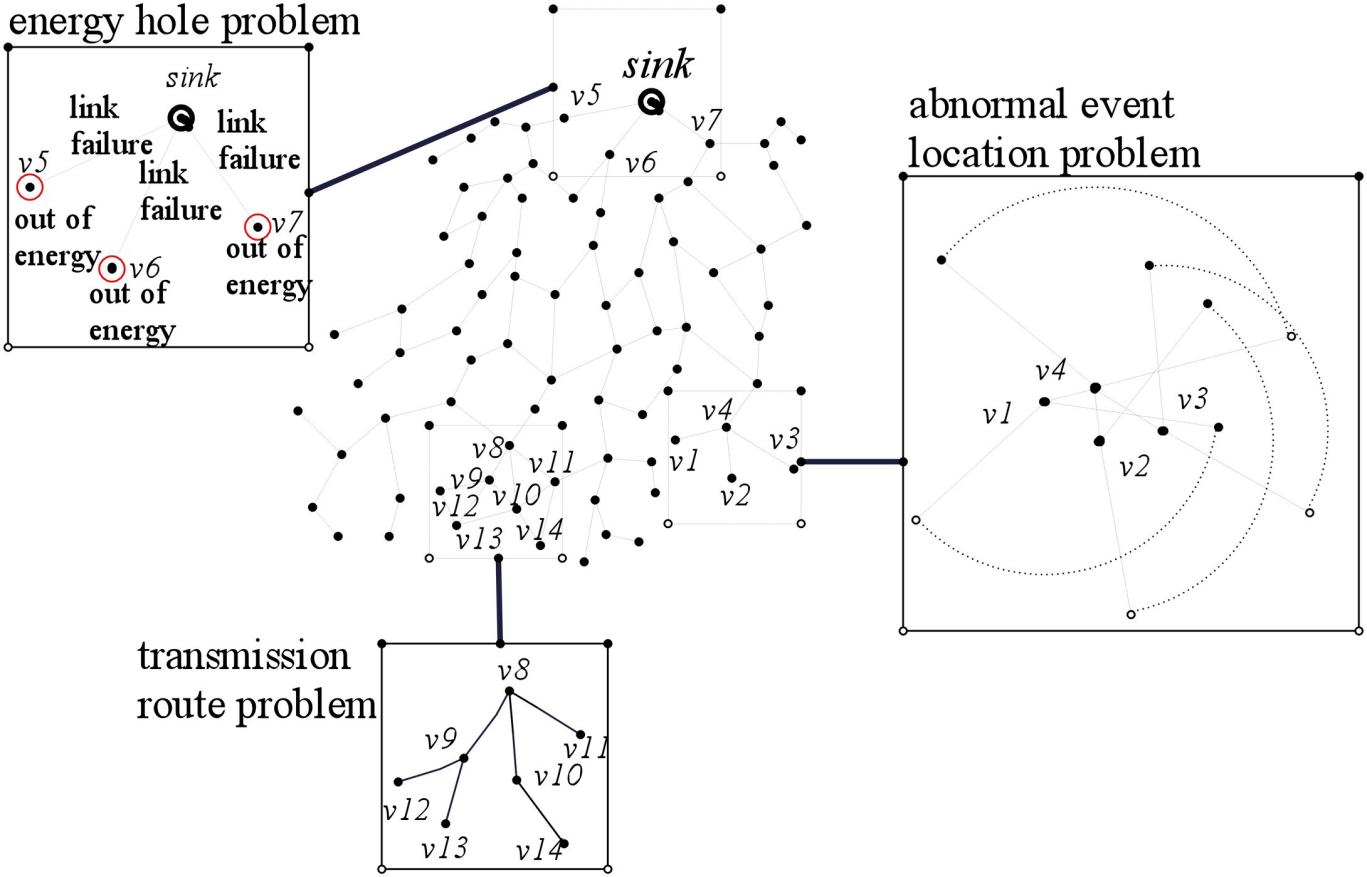
A visual data gathering network system of an LVSN. Three main factors affect the network lifetime: 1) Locating the abnormal event is a challenging and vital matter for prolonging the network lifetime. 2) Energy holes pose a great risk of reducing the lifetime of LVSNs. 3) The transmission route is typically associated with the network lifetime in LVSNs.

Large-Scale Visual Sensor Networks (LVSNs) is formed by a large amount of spatially distributed low-power visual sensors, which are usually deployed in an area of interest for monitoring particular information via video [1]. LVSNs are widely used in many fields such as visual data surveillance for the earthquake, forest fire and other disasters [2]. Visual data are collected from networked smart distributed visual sensors, processed collaboratively, and transmitted to the sink, which is a control center [3]. An example of visual data gathering network system in LVSN is illustrated in Fig 1. The visual sensors construct a complicated mesh network topology. Each visual sensor communicates with each other within a limited transmission range. It has a camera component to capture the video and a processing component to compress the video. For instance, assume we have four visual sensors *v*1, *v*2, *v*3 and *v*4 in the system. Each of them monitors some certain areas by recording video. The videos captured and encoded at *v*1, *v*2, *v*3 are transmitted by *v*4 to the sink for further analysis.

Since LVSNs represent networks of embedded sensors and processors with tightly limited resources [4], more challenges appeared in LVSNs comparing to the conventional sensor networks [5]. Arm to evaluate the performance of the network architecture designed for LVSNs, the network lifetime is always considered as one of the crucial metrics. In this paper, a comprehensive visual data collection network architecture is designed. The contributions of this paper are listed as follows.

1. One Comprehensive Visual Data Gathering Network Architecture (CDNA) is proposed, which considers three main problems comprehensively.
  Problem 1. The abnormal event location is a challenging and vital problem for prolonging network lifetime of LVSNs. In general, lots of energy is wasted at many sensors when no event can be monitored. If a mechanism is appropriately designed, the sensors are allowed to monitor the environment, alternately, for prolonging the network lifetime. Furthermore, the monitoring activities are heavily driven by the appearance frequency of the abnormal event depending on the space and the time. An example is shown in Fig 1. When there is no abnormal event needs to be monitored in the area covered by visual sensors *v*1, *v*2, *v*3 and *v*4, these sensors can be pushed into the sleeping mode for the energy saving purpose. Otherwise, these four sensors can be pulled back to the active mode in rotation.
  Problem 2. Energy hole problem is a risk factor for reducing network life of LVSNs. The energy hole problem occurs when the connectivity of the LVSN cannot be guaranteed. In another word, the communication path from the data source to the control center cannot be constructed, thus the network lifetime is over after all paths disappear [6, 7]. An example of an energy hole is shown in Fig 1. When sensors *v*5, *v*6 and *v*7 run out of energy, the link failure appears between them and the sink. Thus, the communication path to the sink is down and poses considerable risk of reducing the network lifetime.
  Problem 3. The transmission route is typically associated with the network lifetime in LVSNs. The energy consumption for packets gathering and transmitting are higher than conventional sensor networks. An example is shown in Fig 1. Many links exist among sensors *v*8, …, *v*14. Thus, provide efficient transmission routes can save the energy while transmit the visual data successfully.
2. To resolve the first problem, a novel *α*-hull based event location algorithm, which is oriented from the geometric model of *α*-hull, is designed for accurately and efficiently detect the location of the event.
3. Energy hole problem mainly coursed by the unbalanced deployment of the visual sensors. The suitable deployment of the visual sensors can alleviate the energy hole problem. The Chi-Square distribution event-driven gradient deployment method is proposed to reduce the unbalanced energy consumption for alleviating energy hole problem.
4. For solving the third problem, an energy hole repairing method is designed. Firstly, an efficient data gathering tree algorithm is proposed for forwarding the visual data to satisfy the basic premise of the data aggregation. Secondly, since there are lots of redundant sensors in LVSNs, a movement algorithm is designed by moving the redundant sensors to the energy holes areas, which can prolong the network lifetime by repairing the energy holes.

The rest of the paper is organized as follows: the related works in prolonging the network lifetime and the abnormal event allocation are reviewed in Section 2. The performance models are defined in Section 3. The proposed CDNA is presented in Section 4. The simulation results and the discussions are given in Section 5. Finally, the paper is concluded in Section 6.

## Related works

### Network lifetime

In the previous researches, network lifetime issue has been caused wide public concern [7–15]. Such as network construction framework has been provided, which is by modeling the sensors’ roles according to the special abilities, special tasks and time periods [10]. Ugur et al. [11] indicate that two factors of the forwarding packet size and the transmission energy consumption have important effect on prolonging the network lifetime of sensor networks. By considering the forwarding packet size and the forward energy consumption, one integer linear programming based lifetime maximization framework has been proposed. Lifetime maximization for sensor networks using an optimal routing approach was studied in [13]. However, since the difference between the conventional sensors and the visual sensors, these frameworks cannot be applied directly to the WVSN.

As a hot topic, WVSN has became a research issue of great interest [1–5, 16, 17]. For example, minimizing the video distortion by optimizing the power allocation in visual sensor networks was investigated in [16]. Different from the optimization in the conventional wireless sensor network environments [14], He et al. propose a lifetime maximization strategy for WVSN, which consider the source data rates, the encoding method of the data package, and the design of forward routing [1]. Based on the signal strength difference between the normal sensors and the visual sensors, the optimization combined by central coordination and distributed scheme is proposed in [2]. Jiang et al. [17] investigate the effect of WVSN topology and utilize the shortest path algorithm to the graph for video data transmission over visual sensor networks. However, these methods are not suitable for large-scale networks since the situation in large-scale networks is much more complicated. Considering only a few aspects is not easy to solve the problems in the LVSNs. To overcome the limit built by existing methods, a comprehensive visual data gathering network architecture is proposed in this work.

### Abnormal event location

The abnormal event location is one of the critical issues in visual data gathering network architecture of LVSNs [18]. For example, when forest fire occurs, the sensors can only send back the detected event after the source location is located. The earlier the location is identified, the greater the chance that the trapped people can be rescued. Furthermore, property loss and the environment damage can be reduced. Hence, locating events within a margin of error brings in a significant interest of researchers.

The abnormal event localization methods can be divided into subdivisions of anchor-node and the anchor-free. For the anchor-node methods, Chaurasiya et al. [19] conclude them into two kinds of generic methods: the range-based and the range-free. Both methods require the support of the anchor nodes, which are the sensors equipped with the GPS unit to provide the reference position information. Nevertheless, the cost of a sensor module with the GPS unit could be ten to hundreds of times more than the one without the GPS unit. This reality makes it unrealistic to increase the number of anchor sensors in the large-scale sensor networks for the application of affordable disaster management [20]. Hence, the anchor-free methods are more suitable for LVSNs.

For the anchor-free methods, Wang et al. [21] propose a source protected protocol in the phantom routing with the locational angle. The inclination angle is used to guide the random walk processes to avoid choosing the harmful paths to the privacy of the source location. However, a single source is not enough for the monitoring event application. A weighted subtractions negative add on positive multi-source location algorithm was applied to localize the multiple sources by Cheng et al. [22]. Nevertheless, the computational complexity of their method is high and thus is not suitable for LVSNs. In order to prolong the sensor network lifetime, a secondary cluster aggregation with the energy-based and the location-based method is proposed [23]. Moreover, based on the cooperative scheme and mean-field variational inference, the positioning algorithm is proposed in [24]. In this algorithm, they consider the uncertainty of measurement accuracy and the effect of different surrounding environments. In the same year, Nasseri et al. [25] propose an algorithm called the time-bounded re-localization algorithm for the mobile sensor network. In order to examine the characteristic of overlapping in visual monitoring systems, and according to the different sensor parameters and the regional characteristics of the monitored area, a robust dynamic programming framework is proposed by using a deterministic modeling approach [26]. However, the existing methods are for the small-scale visual sensor network environment, and they do not consider the higher visual data throughput in LVSNs. To fill up the gap between the existing methods and the need for large-scale applications, a more suitable abnormal event location algorithm need to be designed in LVSNs.

## System models

As discussed in Section 2 (Related works), the primary research trend has just recently focused on one or two aspects of the network lifetime maximization in LVSNs. A fundamental problem here is to construct a comprehensive visual data gathering network architecture while satisfying various functional and resource requirements. Three main problems are considered comprehensively: (i) to design an abnormal event location algorithm for active visual sensor subset selection, which is able to monitor the area of interest sufficiently, (ii) to design a sensors deployment method for alleviating energy hole problem caused by unbalanced loading appropriately, and (iii) to design an energy hole repairing method and a data transmission route algorithm for resolving the unbalanced load and energy hole problem. In this section, we describe the network graph, the energy consumption model, the definition of network lifetime, and give the geometric model for the abnormal event location. These models and definitions are used to construct comprehensive visual data gathering network architecture in the next sections.

### Network graph

In this paper, we assume that all visual sensors in the LVSN are all equipped with the homogeneous wireless communication model, which allows them to transmit/receive data from any direction within the coverage radius. A total number of *n* sensors are deployed in the simulation area. A LVSN can be modeled as a connected bidirectional graph G=(V,E), where V is the set of the sensors and E is the set of edges. For each edge (u,v)∈E, *u* can be defined as a member of the *v*’s 1-hop neighbor set [27]. Furthermore, each visual sensor can be taken to where it’s needed by person or vehicle.

The connectivity is an indivisible factor for applications among sensors in the multi-hop wireless network, especially, in LVSN. The connectivity in LVSN depends on the reachable neighborhoods within the communication range [28]. It implies that the connectivity reduces to zero when sensors cannot find available neighborhood to transmit the data. In such a case, the energy hole appears and affects both the coverage and the connectivity of the LVSN.

**Definition 1**. **Energy hole** [7]: The energy hole appears when sensors cannot find their neighborhood for forwarding data. As a result, the entire network is subject to premature death because the energy hole separates it into disjoint units.

### Energy consumption model

Calculating the energy consumption of the energy-constrained LVSN is one of the approaches to estimate and manage the network [19] effectively. To reduce the computational complexity, all sensors are assumed to have the same transmission radius *R*_*t*_ and the initial energy *E*_0_ except an infinite energy source is allocated to the sink of the LVSN. Since the LVSN has many redundant visual sensors in the field, the sensors are assumed to operate in either the active mode or the sleeping mode. We do not consider the energy consumption in the sleeping mode because it is small enough to be neglected [7] while Eq (1) can calculate the energy consumption of an active sensor *i*.

**Definition 2**. **Energy consumption** [7]:

$$\begin{array}{c} E_{i}=a_{1}\times E_{i}^{t}+a_{2}\times E_{i}^{r}+a_{3}\times l_{i}\times E^{m} \end{array}$$ (1)

where $E_{i}^{t}$ and $E_{i}^{r}$ represent the energy cost of transmitting and receiving the data by sensor *i*, respectively. *a*_1_, *a*_2_ and *a*_3_ are influence coefficients. Since the movement algorithm is designed for repairing the energy holes, some sensors can be moved by vehicle, then we assume *l*_*i*_ is the displacement distance where sensor *i* moved, *E*^*m*^ is the energy cost of moving the sensor.

### Network lifetime

In a small-scale visual sensor network, each area is monitored by a visual sensor. The exhaustion of energy at any sensor causes the failure of the whole network. The whole system loses its function even most of the visual sensors are still functional. The network lifetime in such applications is defined as the minimum sensor’s lifetime [1, 29]. However, this kind of definition is not suitable for the LVSNs, because the total number of visual sensors in the network is too large. Instead of using the minimum sensor’s lifetime, the connectivity is considered for defining the network lifetime in this paper. Only the sensors in the active mode are processing the monitoring and transmission tasks. These sensors and the transmitting route construct a subgroup *G*_*j*_ of the whole LVSN *G*. The Network lifetime is defined as Definition 3.

**Definition 3**. **Network lifetime**: The network lifetime is defined as slotted into a large number of subgraph time periods [*t*_1_, *t*_2_, …]. When the subgroup *G*_*j*_ is disconnected at time *t*_*j*_, another connected subgraph *G*_*j*+1_ should be constructed to replace it. Perform this process repeatedly, until none of the connected subgraphs can be constructed. In other words, when the remained sensors cannot guarantee network connectivity, the visual data cannot be transmitted to the sink successfully. The network lifetime of LVSN is given by Eq (2).

$$\begin{array}{c} T=\underset{\left(j=1,2,\ldots \right)}{⋃}T_{j}\; \end{array}$$ (2)

where *T*_*j*_ = [*t*_*j*−1_, *t*_*j*_] is the active time interval of subgroup *G*_*j*_.

### Geometric model for event monitoring

One of the geometric models, convex hull, will be used as the foundation of the event monitoring. As a particular convex hull, *α*-hull presents better quality in general. Thus, the concepts of convex hull and *α*-hull will be reviewed briefly below.

**Definition 4**. **convex hull** [30]: Given a set $P=\{p_{0},p_{1},\ldots ,p_{n}\}$ and a point *p*, the convex hull of the set P is denoted by conv(P), where $conv\left(P\right)=\left\{\sum_{i=1}^{n}\alpha_{i}p_{i}|p_{i}\in P,\sum_{i=1}^{n}\alpha_{i}=1,\alpha_{i}\geq 0,i=1,\ldots ,n\right\}$.

**Definition 5**. ***α*-hull** [31]: For the real numbers *α* and a finite set of points P in a plane, the intersection of all the *α*- disks that inclusive P defined as the *α*-hull of P. All the points on the border of the *α*-hull defined as the *α*-hull vertex of the set P.

For *α* < 0, the *α*-hull of P is the complement of the union of all disks of radius −1/*α* that do not contain any point of P. For *α* = 0, the *α*-hull is just the convex hull, and for *α* > 0, the *α*-hull of P is the intersection of all disks of radius 1/*α* that contain P.

**Fig 2:**
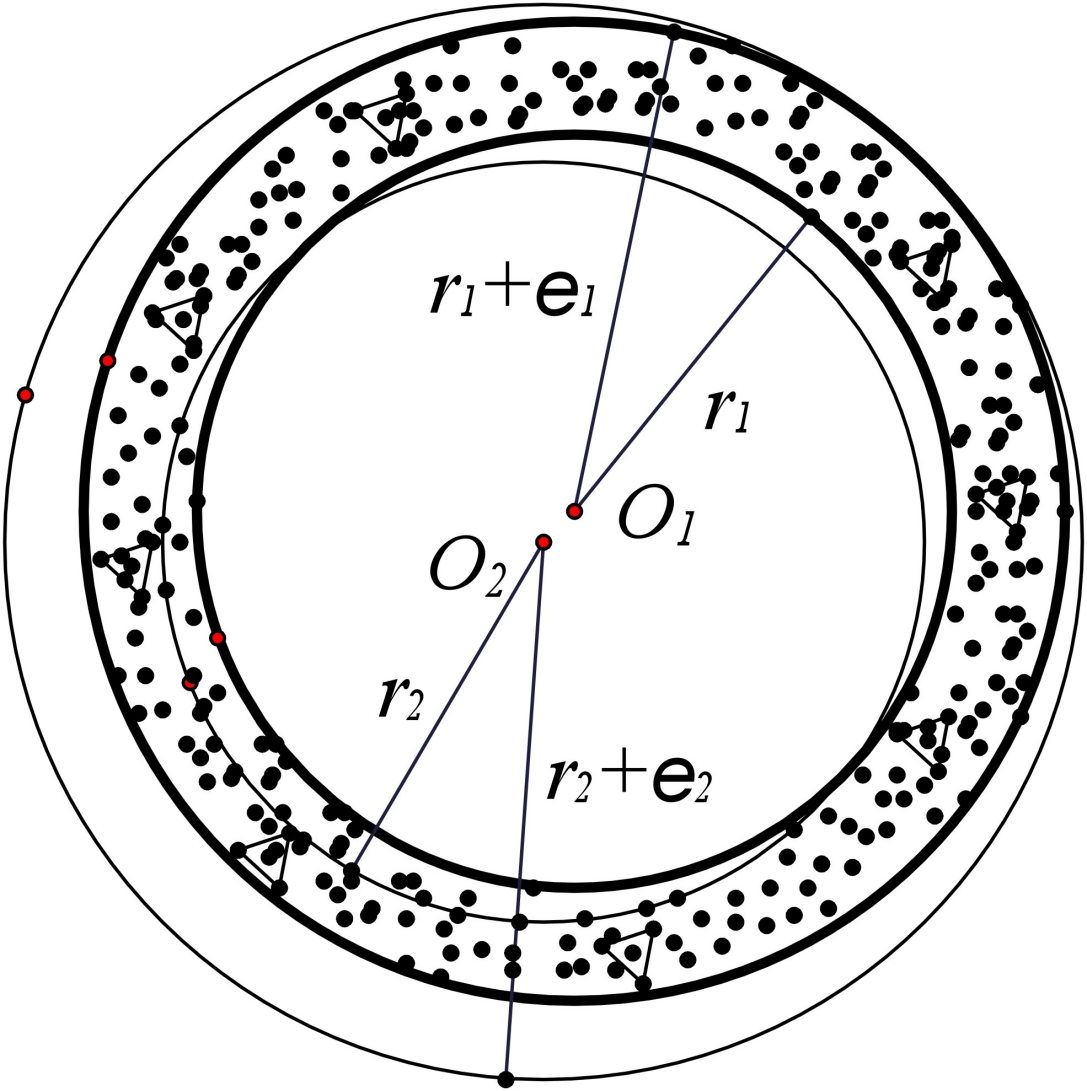
The minimize area circle for set P: The triangles are the sampling point set $P=\{p_{0},p_{1},\ldots ,p_{n}\}$. The concentric circles *C*_1_ and $C_{1}^{\prime }$, with the center point *O*_1_, are capable of containing all the points in set P. The radiuses of *C*_1_ and $C_{1}^{\prime }$ are *r*_1_ and *r*_1_ + *ε*_1_, respectively. The radius of the maximum inscribed circle *C*_2_ is *r*_2_, and its center point is *O*_2_. The shortest distance of set P from *O*_2_ is *r*_2_ + *ε*_2_.

As shown in Fig 2, assume the triangles are the sampling point set $P=\{p_{0},p_{1},\ldots ,p_{n}\}$. According to the minimize area circle constructed method, the concentric circles *C*_1_ and $C_{1}^{\prime }$, with the center point *O*_1_, are capable of containing all the points in set P. The radiuses of *C*_1_ and $C_{1}^{\prime }$ are *r*_1_ and *r*_1_ + *ε*_1_, respectively. The radius of the maximum inscribed circle *C*_2_ is *r*_2_, and its center point is *O*_2_. The shortest distance of set P form *O*_2_ is *r*_2_ + *ε*_2_.

**Lemma 1** [31]: For the minimum area circle *C*_1_, which contains all the points in set P. There are *r*_1_ + *ε*_1_ ≤ *r*_2_ + *ε*_2_ and *r*_1_ ≥ *r*_*m*_ − *ε*_*m*_. The radius and the center of the minimum circumscribed circle *C*_*m*_ and *O*_*m*_ can be calculated by iteration. The shortest distance of set P from *O*_*m*_ is *r*_*m*_ − *ε*_*m*_.

The event monitoring algorithm is proposed based on the **Lemma 1** in the next section.

## A Comprehensive Visual Data Gathering Network Architecture (CDNA)

As discussed in Section 1 and the example shown in Fig 1, we conclude that three aspects will affect the network lifetime: i) event location by the visual sensors, ii) energy hole problem coursed by the unbalanced deployment of visual sensors, and iii) the transmission route problem. In this section, these problems are described in details, and the solutions are proposed.

Firstly, it is entirely waste for the limited energy if all sensors transmit their visual data to the sink for the same detected event. A large quantity of facts has proved that only abnormal event information is significant. It implies that the visual sensors, which are located in the abnormal event area need to be set as the active mode while others can be set as the sleeping mode for the energy saving purpose. To achieve this purpose, we need to propose an abnormal event location algorithm for the active visual sensor subset selection. It enables monitoring the area of interest sufficiently while energy saving is also taken into account at the same time.

Secondly, since several visual data need to be transferred to the sink, those sensors near the sink will eventually die out earlier than the others because of those sensors carry heavier data forwarding load. When those sensors around the sink die out completely, the connectivity of the network can not be guaranteed, and the energy hole appears. Thus, a sensor deployment method need to be designed for alleviating energy hole problem caused by unbalanced loading appropriately.

Thirdly, sensor deployment method can alleviate the energy hole problem in a certain degree, but it can not solve the problem completely. Hence, an efficient energy hole repairing method need to be proposed. On the other hand, limited by the energy source, how to forward the data of the detected events efficiently is a challenging problem in LVSN. To come up with a solution, the data transmission routing algorithm need to be designed for prolonging the network lifetime.

### A Comprehensive Visual Data Gathering Network Architecture (CDNA)

According to the analysis, to achieve the maximum network lifetime for LVSN, a Comprehensive Visual Data Gathering Network Architecture (CDNA) is proposed in this section. The CDNA can be depicted in three steps: 1) The abnormal event location algorithm: an Event Location Algorithm based on the *α*-hull (*α*-ELA) is designed to determine locations of the event efficiently and accurately; 2) The sensor deployment method: a Chi-Square distribution Event Driven Gradient Deployment method (*χ*^2^-EDGD) is designed for alleviating energy hole problem; 3) The energy hole repairing method: The energy hole repairing is considered in two steps. Firstly, an Efficient Data Gathering Tree (EDGT) algorithm is proposed for forwarding the visual data, which are the basic premise of the data aggregation. Secondly, a Movement Algorithm (MA) is designed for solving the energy hole problem by moving the redundant sensors to the energy holes areas.

### Event location algorithm based on *α*-hull (*α*-ELA)

**Fig 3:**
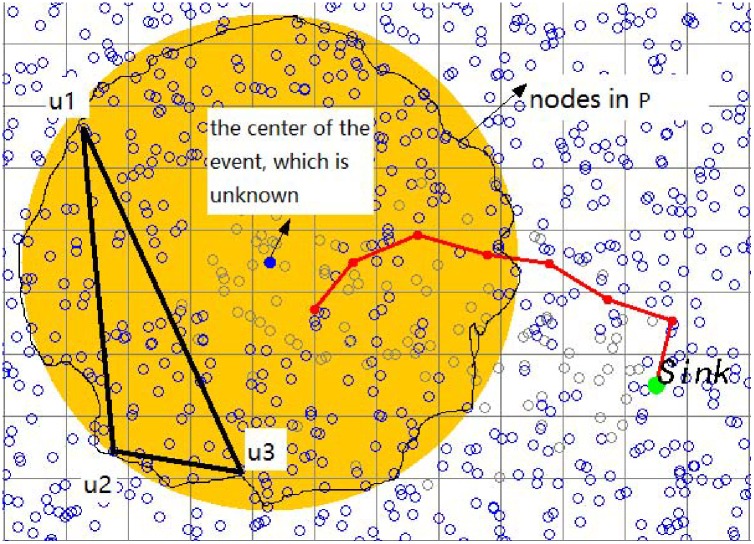
One example of event location algorithm based on *α*-hull: Those sensors at the marginal area of the yellow circular are defined as the set P. Three sensors *u*_1_, *u*_2_, *u*_3_ are selected randomly into the set $P_{1}$ initially. After execute *α*-ELA algorithm, the kindling point will be located.

An example is shown in Fig 3. Assume that there is a fire accident at the center of the yellow circle. The visual sensors, whose sensing distance is less than 200 units, can monitor the accident. There may be too much smoke to confirm the actual kindling point. The visual sensors in the yellow circular internal region in Fig 3 are considered can monitor this event. The actual kindling point need to be confirmed by these visual sensors’ positions. Thus, an Event Location Algorithm based on *α*-hull (*α*-ELA) is designed.

According to **Lemma 1** and the principle described as follows. If the points set P is contained by the concentric circles $A=\{C_{0},C_{1},\ldots \}$, the inside and outside circles’ radius are *r* and *r* + *ε*, then the minimum area circle *C*_0_, whose inside circle can get through two points of the set P’s *α*_1_-hull $\left(\frac{1}{\alpha_{1}}=\varepsilon -r\right)$. A’s outside circle can get through two points of the set P’s *α*_2_-hull $\left(\frac{1}{\alpha_{2}}=r+2\varepsilon \right)$.

**Algorithm 1**: Event Location Algorithm based on the *α*-hull (*α*-ELA)

**1** $P=\{p_{i},i=1,2,3\ldots \}$; $P_{1}=\left\{p_{1},p_{2},p_{3}\right\}$;

**2** call function *f*(*p*_1_, *p*_2_, *p*_3_) by Alg. 2; $r_{f}=\max\;d\left(P_{1},O\right)$, $r_{c}=\min d\left(P_{1},O\right)$, *r* = *r*_*f*_;

**3 while** ($P_{1}\neq P$) **do**

**4**  **if** (*r*_*c*_ ≥ *r*_*f*_) **then**

**5**   *r* = *r*_*c*_; break;

**6**  **end**

**7**  **else**

**8**   *α* = 1/*r*;

**9**   $P_{1}=$ {sensors on the *α*-hull};

**10**  **end**

**11**  **for** *(*$\forall p_{i}\in P$*)* **do**

**12**   call function *f*(*p*_1_, *p*_2_, *p*_*i*_) by Alg. 2;

**13**   **if** *(O* ∈ {*α* − *hull*}*)* **then**

**14**    break;

**15**   **end**

**16**   **else**

**17**    find three points *p*_1_, *p*_2_, *p*_3_, who construct the biggest obtuse angle of the triangle;

**18**    call function *f*(*p*_1_, *p*_2_, *p*_3_) by Alg. 2;

**19**   **end**

**20**   $P_{1}=P_{1}⋃\left\{p_{i}\right\}$;

**21**   *r* = (*r*_*c*_ + *r*_*f*_)/2; *r*_*c*_ = *r*;

**22**  **end**

**23 end**

**24** return (evernt location and radius $L_{s_{i}}=O,r$);

The *α*-ELA algorithm can use the iteration method. Initially, three non-collinear points $P_{1}=\left\{p_{1},p_{2},p_{3}\right\}$ are selected from the set P (Line 1 in Alg. 1). Calculate the center *O* of the circle cross the set $P_{1}$, and calculate the radius of the minimum circumscribed circle *r* (Lines 2-6 in Alg. 1). Then, find out the *α*-hull of the set $P_{1}$, and update the set $P_{1}$, and those sensors on the *α*-hull are added into the set $P_{1}$ (Lines 8-9 in Alg. 1). If all sensors are in the same circle, calculate the center and the radius of the circle (Lines 12-15 in Alg 1). Otherwise, find out the biggest obtuse angle of the triangle, and the edge which obtuse angle corresponding to is the diameter of the minimum circumscribed circle, then call function *f*(*p*_1_, *p*_2_, *p*_3_) by Alg. 2 to calculate the center and the radius of the circle (Lines 16-19 in Alg. 1). This progress adjusts the center of the minimum circumscribed circle and the radius by Lemma 1 (Lines 11-22 in Alg. 1).

Alg. 2 is a function to calculate the center *O* and the radius *r*. The main method is that the center is in the intersection of the mid-perpendicular of each edge. Initially, the three points are passed by the main program (Lines 2, 12 and 18 in Alg. 1). Find the slope and midpoint of any two points. The negative inverse of the slope is the slope of the perpendicular bisector. The mid-perpendicular can be calculated by the slope and midpoint (Line 2 in Alg. 2). Then, the intersection can be found from the two perpendicular bisector equations, which is the center *O* (Lines 3,4 in Alg. 2). The radius *r* can be calculated (Line 5 in Alg. 2).

**Algorithm 2**: Calculating the center *O* and the radius *r* (function *f*(*p*_1_, *p*_2_, *p*_3_))

**1** three points *p*_1_ = (*x*_1_, *y*_1_), *p*_2_ = (*x*_2_, *y*_2_), *p*_3_ = (*x*_3_, *y*_3_);

**2** *a* = 2 × (*x*_2_ − *x*_1_); *b* = 2 × (*y*_2_ − *y*_1_); $c=x_{2}^{2}+y_{2}^{2}-x_{1}^{2}-y_{1}^{2}$; *d* = 2 × (*x*_3_ − *x*_2_); *e* = 2 × (*y*_3_ − *y*_2_); $f=x_{3}^{2}+y_{3}^{2}-x_{2}^{2}-y_{2}^{2}$;

**3** *x* = (*b* × *f* − *e* × *c*)/(*b* × *d* − *e* × *a*);

**4** *y* = (*d* × *c* − *a* × *f*)/(*b* × *d* − *e* × *a*);

**5** $r=\sqrt{\left(x-x_{1}\right)\times \left(x-x_{1}\right)+\left(y-y_{1}\right)\times \left(y-y_{1}\right)}$;

**6** return (*O* = (*x*, *y*), *r*);

As the example shown in Fig 3, those sensors at the marginal area of the yellow circular are defined as the set P. Three sensors *u*_1_, *u*_2_ and *u*_3_ are selected randomly into the set $P_{1}$ initially. After execute the *α*-ELA algorithm, the center of the fire accident can be located.

### Chi-Square distribution Event Driven Gradient Deployment method (*χ*^2^-EDGD)

The random deployment method does not consider the unbalanced load problem and is not suitable for the visual data gathering application in LVSNs. Since sensors around the sink bear the burden of forwarding information, if higher density sensors are deployed around the sink, the connectivity is guaranteed. The density of the deployment of the sensors can be decreased by the gradient.

Chi-Square (*χ*^2^) distribution deployment can satisfy this requirement. *χ*^2^ distribution has excellent characters, which is suitable for the LVSN deployment. Firstly, there is only one parameter in *χ*^2^ distribution, which is the degree of freedom *k*. That is to say, only one parameter needs to be considered when the sensors are deployed in the LVSNs. In addition, it tends to be normally distributed at large degrees of freedom. Secondly, *χ*^2^ distribution is additive. If some distributions obey the *χ*^2^ distribution, and they are mutually independent. The sum of them is also obeying *χ*^2^ distribution. That is to say when new sensors need a supplement, *χ*^2^ distribution can be used again, which will not change the original network topological property. The density function of *χ*^2^ distribution is given in Eq (3).

$$\begin{array}{c} \varphi_{k}\left(x\right)=\{\begin{array}{c} Ax^{\frac{k}{2}-1}e^{-\frac{x}{2}}\;\;\;\left(0,\infty \right)\\ 0\;\;\;\;\;\;\;\;\;\;\;\;\;\;\;(-\infty ,0) \end{array} \end{array}$$ (3)

where $A={\left(\int_{0}^{\infty }x^{\frac{k}{2}-1}e^{-\frac{x}{2}}dx\right)}^{-1}$, which can guarantee that $\int_{0}^{\infty }\varphi_{k}\left(x\right)dx=1$. *k* is the degree of freedom.

*χ*^2^-EDGD is gradient based, the number of sensors in each local area can be calculated obey *χ*^2^ distribution. The detail of *χ*^2^-EDGD is described in Algorithm 3. The whole network is divided into some local areas, which are according to the distance from the sink (Line 1 in Alg. 3). The proportion for each local area *D*_*i*_ can be calculated according to Eq (3) (Line 2 in Alg. 3). At last, based on the total number of sensors which will be deployed into the network and the rate, the number of the sensors *n* × *D*_*i*_, which will be deployed in the area *S*_*i*_ (Line 3 in Alg. 3).

**Algorithm 3**: Chi-Square distribution Event Driven Gradient Deployment method (*χ*^2^-EDGD)

**1** Divide the target area according to the distance from the sink, each part is recorded as *S*_*i*_;

**2** Calculate *D*_*i*_ and the band of the location;

**3** Calculate the number of the sensors *n* × *D*_*i*_, which will be deployed in the area *S*_*i*_;

**4** Deploy the sensors into each area.

**Fig 4:**
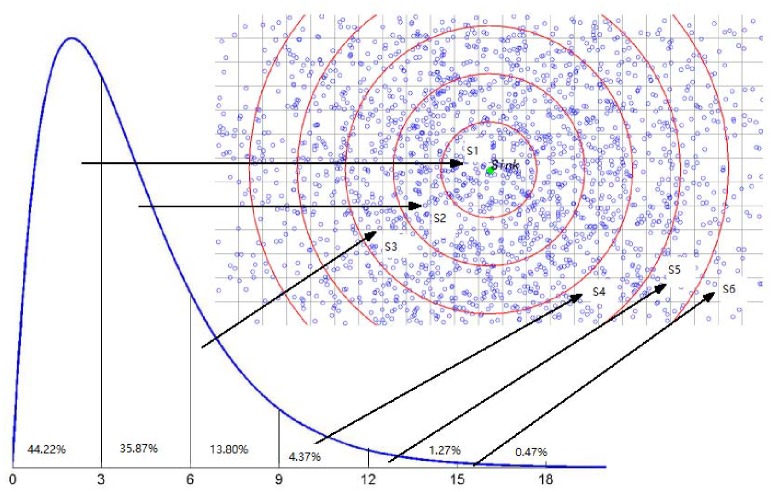
The deployment model by *χ* ∼ *χ*^2^(4) distribution: Assume the network area is divided into 5 parts, *S*_1_, *S*_2_, …, *S*_5_, and *n* sensors will be deployed into the network. By observing the curve in Fig 4, there are $\int_{0}^{3}\varphi_{4}\left(x\right)dx=44.22\%;\int_{3}^{6}\varphi_{4}\left(x\right)dx=35.87\%;\int_{6}^{9}\varphi_{4}\left(x\right)dx=13.8\%;\int_{9}^{12}\varphi_{4}\left(x\right)dx=4.37\%;\ldots$. The largest number of sensors *n* × 44.22% can be deployed into the area *S*_1_, which is the closest to the sink. The least number of sensors *n* × 1.27% will be deployed into the area *S*_5_, which is the farthest to the sink.

An example of the the deployment model with *k* = 4 by *χ* ∼ *χ*^2^(4) is shown in Fig 4. The value of the density is decided according to the distance between the range location and the sink node, then the gradient decreasing along the sensors density can be guaranteed. Assume the network area is divided into 5 parts, *S*_1_, *S*_2_, …, *S*_5_, which are some concentric annuluses centered at the sink with the same width. *n* sensors will be deployed into the network. By observing the curve in Fig 4, there are $\int_{0}^{3}\varphi_{4}\left(x\right)dx=44.22\%;\int_{3}^{6}\varphi_{4}\left(x\right)dx=35.87\%;\int_{6}^{9}\varphi_{4}\left(x\right)dx=13.8\%;\int_{9}^{12}\varphi_{4}\left(x\right)dx=4.37\%;\ldots$. The largest number of sensors *n* × 44.22% can be deployed into the area *S*_1_, which is the closest to the sink. The least number of sensors *n* × 1.27% will be deployed into the area *S*_5_, which is the farthest to the sink.

### Efficient energy hole repairing method

For sensors with limited energy sources, how to repair the energy holes by the redundant sensors is a challenging problem in LVSNs. An Efficient energy hole Repairing Method (ERM) with two steps is designed as follows. Firstly, an Efficient Data Gathering Tree (EDGT) algorithm is proposed for forwarding the visual data, which is the basic premise of the data aggregation. Secondly, a Movement Algorithm (MA) is designed for solving the energy hole problem by moving the redundant sensors to the energy holes areas.

The basic routing is designed based on the flooding method, which starts from the sink and tries to find the first hop by the transfer radius and then finding the second hop by the first hop’s sensors transfer radius. The remaining is processed by repeating the procedures mentioned above, flooding to the whole network, and the data collection Tree can be constructed ultimately.

**Fig 5:**
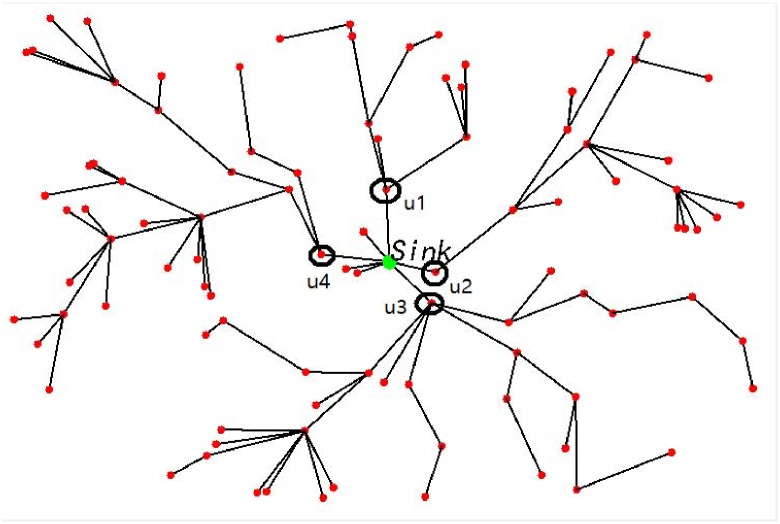
One example of basic routing: 100 sensors aredeployed by the random deployment method in the area and it is assumed that no event occur at the beginning. The aggregation tree among the sensors and the sink can be constructed. It is easy to see that the sensors *u*1, *u*2, *u*3 and *u*4 are only one-hop away from the sink, which carry heavier forwarding load than other sensors.

One example is given in Fig 5: 100 sensors are deployed by the random deployment method in the area, and it is assumed that no event occurs at the beginning. The aggregation tree among the sensors and the sink can be constructed. It is easy to see that the sensors *u*1, *u*2, *u*3 and *u*4 are only one-hop away from the sink, which carries heavier forwarding load than other sensors. The *χ*^2^-EDGD allows the number of forwarding sensors to increase. However, the energy is still wasted even if the number of forwarding sensors is increased. Thus, the EDGT is designed in Alg. 4 to relieve this problem.

**Algorithm 4**: Efficient Data Gathering Tree (EDGT) algorithm

**1** $N_{hop}\left(v_{i}\right)=-1,v_{i}\in V$; T=ϕ; $L(v_{i})=\phi$; $V^{\prime }=\phi$; *R*_*t*_; *R*_*s*_;

**2 for** *(*$v_{i}\in V$*)* **do**

**3**  **if** *(δ*(*v*_*i*_, *sink*) ⩽ *R*_*t*_*)* **then**

**4**   $L\left(v_{i}\right)=L\left(v_{i}\right)\cup \left\{\left(v_{i},sink\right)\right\}$;

**5**   *N*_*hop*_(*v*_*i*_) = 1; $V^{\prime }=V^{\prime }\cup \left\{v_{i}\right\}$

**6**  **end**

**7 end**

**8 while** *(*$V/V^{\prime }\neq \phi$*)* **do**

**9**  **for** *(*$v_{i}\in V/V^{\prime }$ and $v_{j}\in V^{\prime }$*)* **do**

**10**   **if** *(*$\exists v_{k}\in V$ and *N*_*hop*_(*v*_*k*_) = −1*)* **then**

**11**    $\delta \left(v_{k}^{\min},v_{j}\right)=\min\left\{\delta \left(v_{k},v_{j}\right)|v_{k}\in V\right\}$;

**12**    $v_{i}=v_{k}^{\min}$;

**13**    *N*_*hop*_(*v*_*i*_) = *N*_*hop*_(*v*_*j*_) + 1;

**14**    $L\left(v_{i}\right)=L\left(v_{j}\right)\cup \left\{\left(v_{i},v_{j}\right)\right\}$;

**15**    $V^{\prime }=V^{\prime }\cup \left\{v_{i}\right\}$;

**16**   **end**

**17**  **end**

**18 end**

**19 for** *(*∀*s*_*j*_*)* **do**

**20**  **for** *(*$\forall v_{i}\in V$*)* **do**

**21**   **if** *(δ*(*v*_*i*_, *s*_*j*_) ⩽ *R*_*s*_*)* **then**

**22**    $N_{hop}\left(v_{i}^{\min}\right)=\min\left(N_{hop}\left(v_{i}\right)\right)$; $v_{f}=v_{i}^{\min}$;

**23**    $L\left(s_{j}\right)=L\left(v_{f}\right)$;

**24**   **end**

**25**   **else**

**26**    break;

**27**   **end**

**28**  **end**

**29**  $T=T\cup L(s_{j})$;

**30 end**

**31** return T;

Starting from the sink, *N*_*hop*_(*v*_*i*_) is defined as the number of hops between sensor *v*_*i*_ and sink. It is set as *N*_*hop*_(*v*_*i*_) = −1 initially (Line 1 in Alg. 4). At the beginning stage, each sensor *v*_*i*_ need to find its best route to the sink, which is represented by $L(v_{i})$ (Lines 2-18 in Alg. 4). Later on, for each event, *s*_*j*_, the sensor which can monitor the event and has the lowest hops to the sink is selected (Lines 19-30 in Alg. 4). At last, the efficient data gathering tree T can be constructed, and the monitored information can be transmitted to the sink hop by hop through this tree.

In addition, with the basic flooding routing method, all sensors, which detect the occurrence of an abnormal event, transmit the detected event back to the sink. Nevertheless, these multi-transmissions contain many duplicated and redundant information. These multi-transmissions consume energy with no contribution. The best scenario should be that when an event occurs, only one sensor detects it and forward the data packets to the control center. The EDGT algorithm eventually handles the event detection and route construction in this way. Without reducing the coverage of the LVSN, the redundant sensors are set as the sleeping mode while only a few sensors are awake and fully functional. When the awake sensor runs out of its battery, one of the sensor in the sleeping mode is awake to replace the original one and establish a new route for data transmission.

Although the designed deployment method can alleviating the energy hole problem, it can not be resolved thoroughly. There are lots of redundant sensors with low utilization. These sensors can be taken to areas containing energy holes to fill up the vacancy of the dead sensors. Nevertheless, moving the sensors also consumes energy, and the redundant of the original covered area is reduced once the sensor moves away. An effective movement algorithm is essential for maintaining the balance of filling up energy holes and the redundant of the coverage over the whole LVSN.

**Algorithm 5**: Movement Algorithm (MA)

**1 for** *(*∀*s*_*i*_*)* **do**

**2**  $M_{s_{j}}=\left\{l_{1},l_{2},\ldots l_{c}\right\}$;

**3**  **for** *(*∀*v*_*i*_*)* **do**

**4**   **for** *(*$\forall l_{j}\in M_{s_{i}}$*)* **do**

**5**    $\delta \left(v_{i},l_{j}^{\min}\right)=\min\delta \left(v_{i},l_{j}\right)$; $l_{k}=l_{j}^{\min}$;

**6**   **end**

**7**   move to *l*_*k*_;

**8**  **end**

**9 end**

The visual sensors can be taken to the necessary area by some vehicles. One vehicle manages one relatively small area. We expect that only a few vehicles with a shorter movement distance are needed. For this aim, The Movement Algorithm (MA), which is based on a minimum spanning tree and calculus method, is proposed. A line in the space can be represented by a set of equal diversion points when the selected points hold sufficient quantity. The branch, which is equally divided into sections, of the minimum spanning tree between two events can be grown based on the lines formed by the equal diversion points. The proposed MA algorithm is described in detail in Alg. 5. By monitoring the events by Alg. 1, the data gathering tree is constructed for the events by Alg. 4. Then, for each event *s*_*j*_, for the line between the center of *s*_*j*_ and sink, *c* equal diversion points *l*_1_, *l*_2_, …*l*_*c*_ are located, which will be added into the list $M_{s_{j}}$ (Line 2 in Alg. 5). For every sensor, find the closest point *l*_*k*_, and move to it by vehicle (Lines 3-8 in Alg. 5). The MA terminates when either the criteria listed as follows is satisfied: 1) when all sensors become the transmission sensors, which means that they all in the route between the detected event and the sink; or 2) when an existing route already forms the connection between the detected event and the sink.

**Fig 6:**
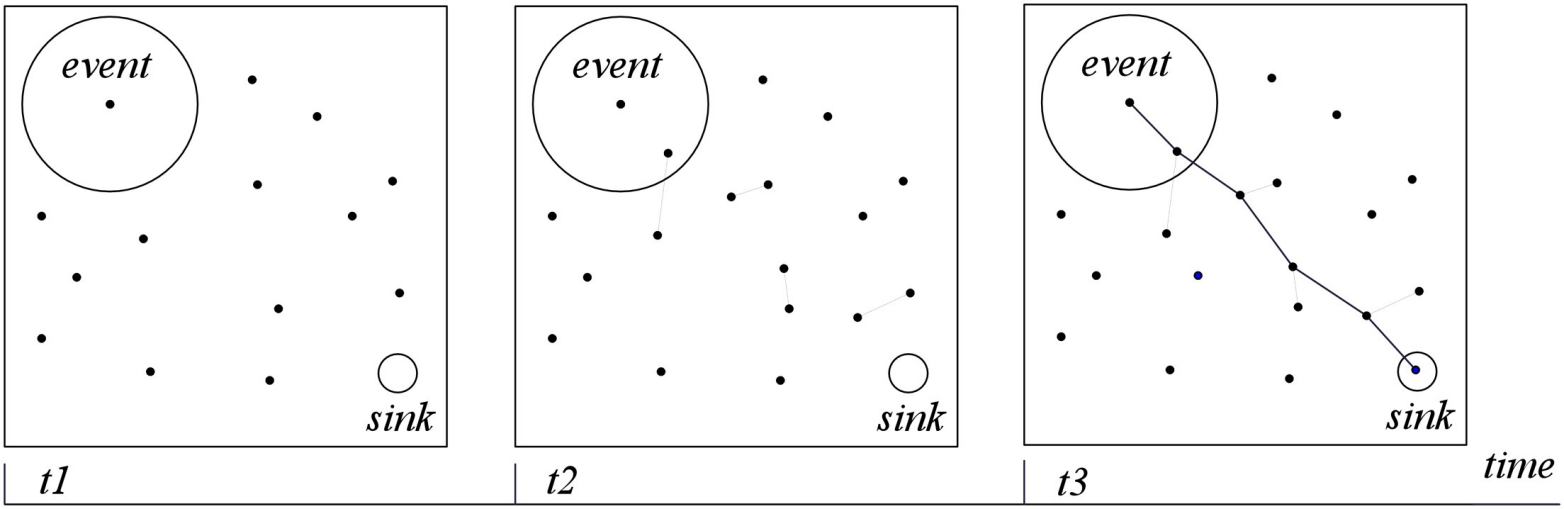
An example of the Movement Algorithm: The horizon axis indicates the time-line. 1) energy hole appears at time *t*_1_, 2) to establish the connection between the detected event and the sink node, the movement algorithm is used at time *t*_2_ (the line between the transmitting sensor in the event occurring area and the sink, is equally divided into 6 sections, 4 equal division points and 2 terminal vertexes are required), 3) the energy holes can be repaired at *t*_3_, and the detected event can be transmitted back to the sink (the proposed MA assigns the vehicle to take the sensors to these 6 points).

One example of the MA is shown in Fig 6. The horizon axis in Fig 6 indicates the time-line. Assume that energy hole appears at time *t*_1_. To establish the connection between the detected event and the sink node, Alg. 5 is used at time *t*_2_ to calculate vehicle mobile path for moving redundant sensors. The line between the transmitting sensor in the area of the event and the sink is divided into 6 equally divided sections, 4 equal division points and 2 terminal vertexes are required. The proposed MA assigns the vehicle to take the sensors to these 6 points. After the movement, the energy holes in the LVSN can be repaired at *t*_3_, and the detected event can be transmitted back to the sink successfully.

## Simulations

In this section, the feasibility and the comparison analysis will be studied by some simulations for evaluating the performance of the algorithms. The simulations are designed to evaluate the performance of algorithms in the environment built by JAVA. By following the common assumptions, the LVSN environment is composed of *n* homogeneous visual sensors with a sink node located at the center of the area. The event monitoring range (*R*_*s*_) is defined as 50 to 100. The visual sensors located in the event monitoring range can pick up the occurrences. The sensor transmission radius (*R*_*t*_) is defined as *R*_*t*_ = 30 for transmitting visual data. Assume *m* events in total occur in the simulation period. The random distribution and the *χ*^2^-distribution are used in the control and the compare simulations, respectively. The sensor distribution presented in the figure is averaged over 50 simulations. The number of sensor nodes deployed in the space is incrementally increased from 500 to 5000 with the step size of 500 sensors. The initial energy of all sensors is assumed to be equal expect the sink node has the infinite energy source. The parameter setting for the simulation is revealed in Table 1.

**Table 1:** Part of the network parameter data.

Parameter	Value
Sensor deployment	Random/ *χ*^2^ distribution
Given Region	1000*1000 *m*^2^
The number of sensors *n*	500-5000
The number of events *m*	1-100
The initial energy of sensors	1 *J*
Sensor transmission radius *R*_*t*_	30*m*
Event monitoring range *R*_*s*_	50*m* − 100*m*
Consumed energy in transmitter circuit *E*^*t*^	0.2 *J*
Consumed energy at the receiver circuit *E*^*r*^	0.1 *J*

### The feasibility of visual data collection network architectures

**Fig 7:**
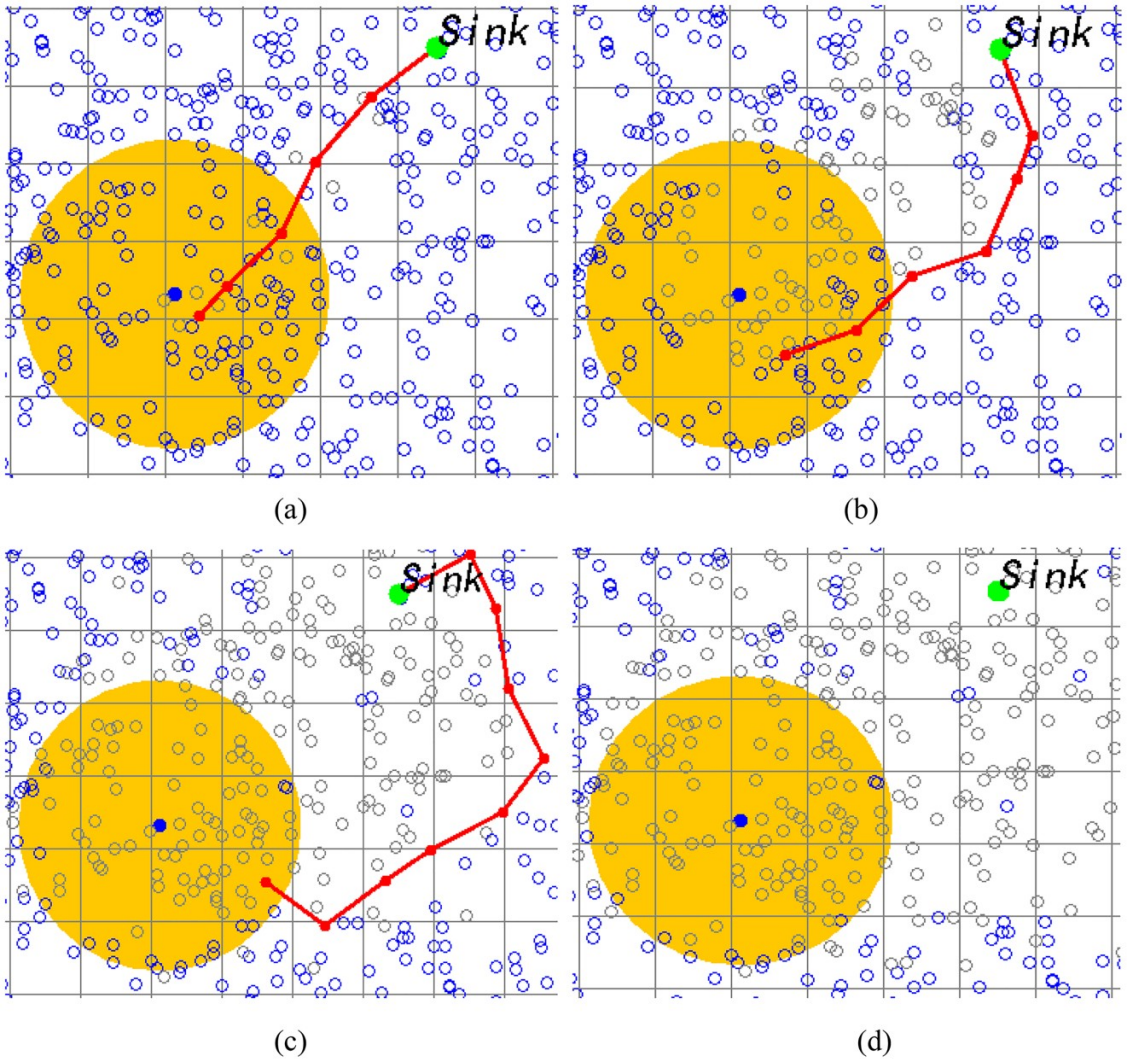
One example of event monitoring with the random distribution deployed LVSN. The light hollow circles, the dark hollow circles and the solid ones are represent the dead, sleep and active sensors, respectively: (a) time *t*_1_; (b) time *t*_2_; (c) time *t*_3_; (d) time *t*_4_.

An example of the event monitoring application with the random distribution deployed LVSN is shown in Fig 7. Assume that the random distribution deploys the sensors, and only one event occur, which need to be monitored by visual sensors around it. By using the *α*-ELA algorithm, the center of the event can be confirmed. Then, the nearest visual sensor detects the occurrence of the event at time *t*_1_, it is defined as the sensing sensor and is responsible for finding the data forwarding route via efficient data collection tree and sending the event information back to the sink (Fig 7(a)). Other visual sensors can be set as the sleeping mode for energy saving. The connectivity of the transmitting route is destroyed when the sensing or forwarding sensors are out of battery. Then, other sensing sensors or forwarding sensors need to be selected for constructing a new route. Such as shown in Fig 7(b) at time *t*_2_ and Fig 7(c) at time *t*_3_. When there is no more available sensors can take up the responsibility, the energy hole appears and is not repairable (Fig 7(d)). The lifetime of the LVSN is terminated because the sink node can no longer receive the detected events.

**Fig 8:**
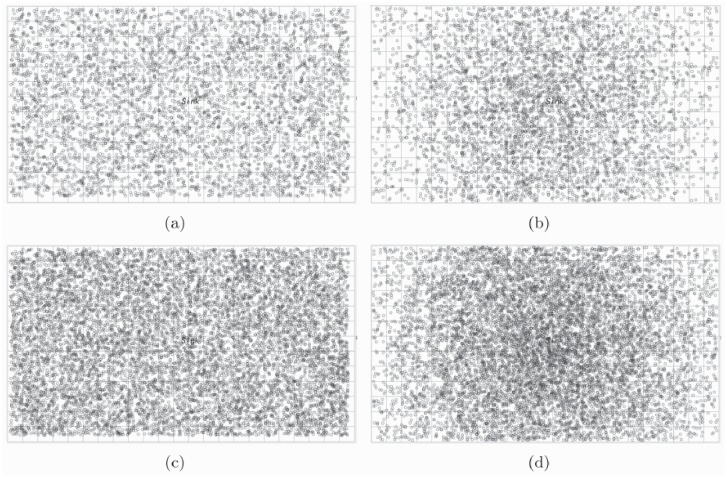
Sensor deployment of LVSN with sink in the center of the network: (a) 5000 sensors deployed by random; (b) 5000 sensors deployed by *χ*^2^ distribution; (c)10000 sensors deployed by random; (d) 10000 sensors deployed by *χ*^2^ distribution.

It is easy to know that those sensors who near the sink will die out early because of the heavier forwarding load. If those sensors around the sink die out completely, the connection of the network can not be guaranteed and the energy hole appeared. The performance of the *χ*^2^-EDGD is given as follows. Assume the sink node is located in the center of the network. Fig 8(a) and 8(b) show 5000 sensors deployed by the random distribution and the *χ*^2^-distribution deployment methods, respectively. Comparing to the same setting but with 10000 sensors deployed in total shown in Fig 8(c) and 8(d), it is obvious that the density of sensors around the sink is much higher in the *χ*^2^-distribution deployment. It implies that with the *χ*^2^-distribution deployment, the system has more redundant paths for relieving the energy hole problem.

**Fig 9:**
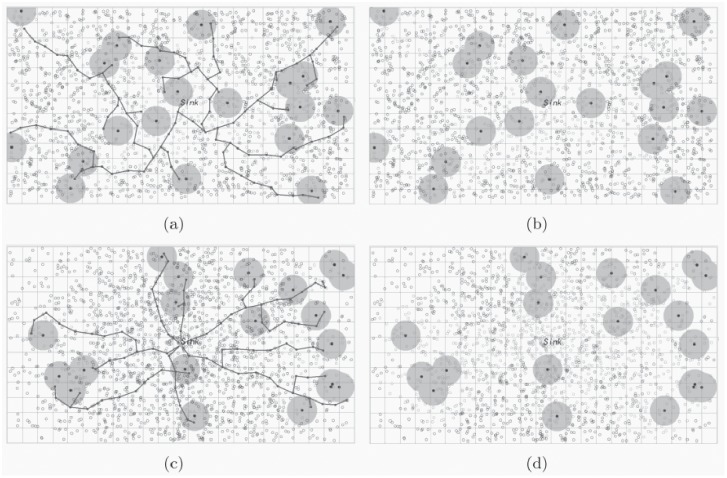
The event monitoring and energy hole problem relieving simulation: (a) The event monitoring and route construction results in the random deployed environment; (b) The network lifetime is over, when the energy hole appears in random deployment environment; (c) The events monitoring in *χ*^2^ distribution deployment environment; (d) The network lifetime is over, when the energy hole appears in *χ*^2^ distribution deployment environment.

The event monitoring and the energy hole problem relieving simulation is shown in Fig 9. In this example, 2000 sensors are deployed in the coverage area. The transmission radius *R*_*t*_ is set to 30 while the total number of the abnormal events is set to 20. The detecting range of the sensor is set to 50, and it can be located by the *α*-ELA. Fig 9(a) shows the event monitoring and route construction results in the random deployed environment. The network lifetime is over when the energy hole appears around the sink as shown in Fig 9(b). The light gray circles represent the dead sensors, and thus there is no route available of linking the events area and the sink.

The same parameter setting applies to the experiment with the *χ*^2^-distribution deployment method. As shown in Fig 9(c) and 9(d), it is observable that the lifetime of the whole network last longer because it can last till much more sensors run out of battery when energy hole appears.

**Fig 10:**
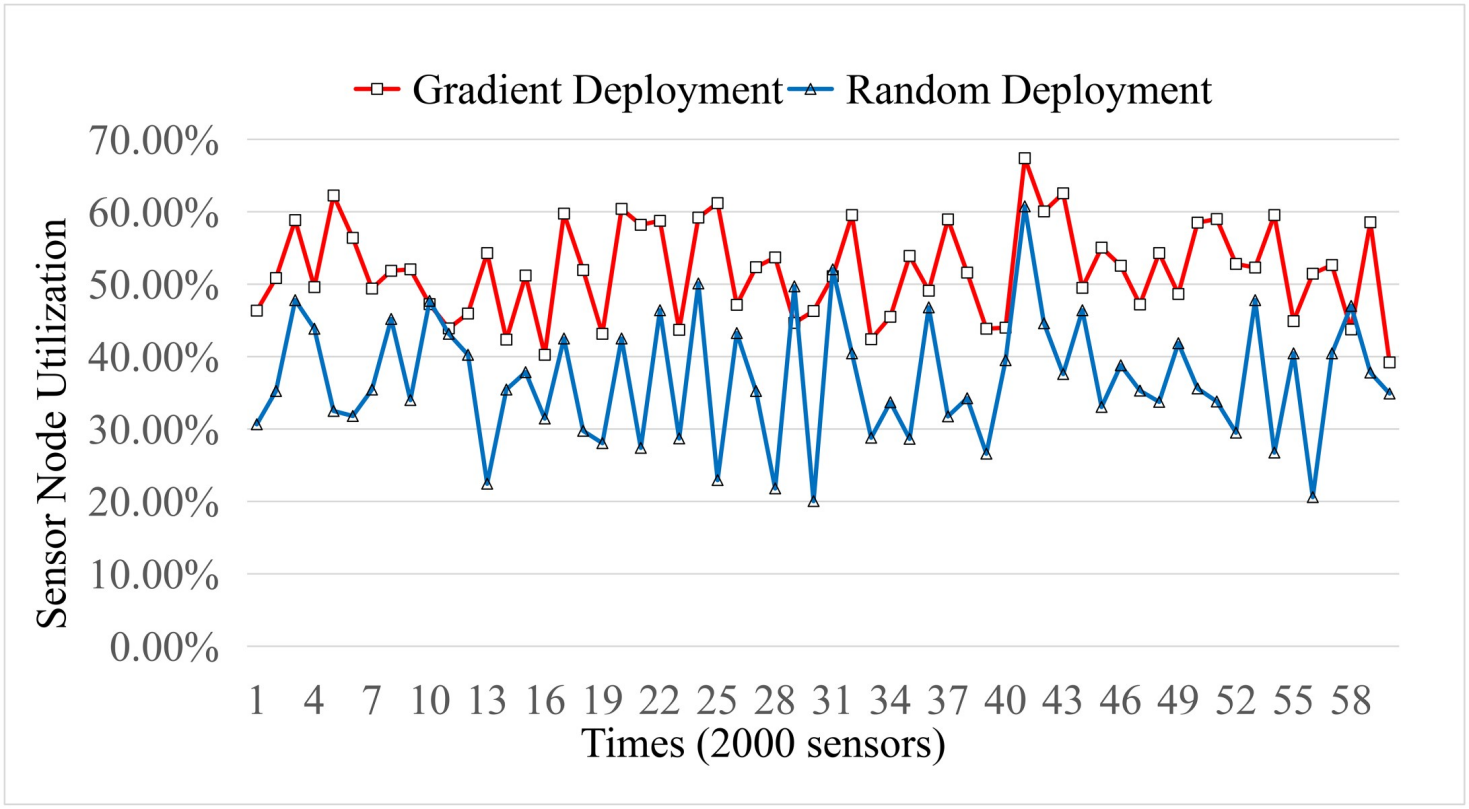
Sensor utilization.

The utilization rate of sensors is revealed in Fig 10. The result is obtained by averaging the results over 50 times of independent run. Although there are some fluctuates in the curve, the general trend can be determined. About 50% sensors are waste by *χ*^2^ distribution deployment, while about 60% sensors are waste by the random deployment. That is to say, *χ*^2^ distribution can relieve the energy hole problem to a certain extent. However, the static sensor is not the best solution for the event monitoring application. The performance can be improved if some redundancy sensors can be taken to the necessary area for event monitoring.

**Fig 11:**
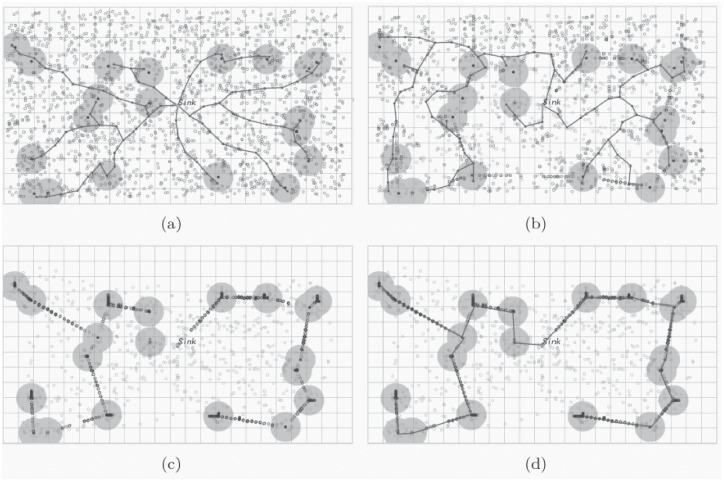
The efficient energy hole repairing method: (a) At time *t*_1_, 2000 sensors are deployed and 20 events are detected in the area; (b) After utilizing the Movement Algorithm to rearrange the redundancy sensors, the energy hole can be repaired and the detected events can be monitored without problems at *t*_2_; (c) Utilizing the Movement Algorithm to rearrange the redundancy sensors at time *t*_3_; (d) Utilizing the Movement Algorithm to rearrange the redundancy sensors at time *t*_4_.

The simulation results of the efficient energy hole repairing method are shown in Fig 11. Fig 11(a) shows that 2000 sensors are deployed and 20 events are detected in the area. The whole network is functional without any energy hole at time *t*_1_. After a period of time, some of the sensors are dead, and finally, the energy hole appears in the system. Nevertheless, after utilizing the Movement Algorithm to rearrange the redundancy sensors, the energy hole can be repaired, and the detected events can be monitored without problems at *t*_2_ as shown in Fig 11(b). Similarly, the results are shown in Fig 11(c) and 11(d), few sensors are wasted.

### The performance of visual data collection network architectures by comparison analysis

Although many researches in WSNs field are focus on network lifetime recent years, the designed evaluation indexes and methods are quite different. For instance, for the evaluation index of monitoring scope, some researches consider the whole sensor network [8], while others consider the range of deterministic assumptions [9]. It is hard to compare the comprehensive architecture with existing methods without uniform indexes [8, 9, 32]. In this paper, we will unify some basic methods for the contrastive analysis. Such as EDGT algorithm will be used as the route method, *α*-ELA abnormal event monitoring method will be used for the monitoring scope. Furthermore, most researches are based on random deployment, for example, Xu et al. propose three random deployment strategies [8]. So our comparative systems are basically to random deployment. Then, four kinds of methods will be compared, 1) the random deployment method without ERM [8, 9], 2) the *χ*^2^-distribution deployment without ERM, 3) the random deployment with ERM, and 4) our proposed CDNA. Before the comparison analysis, the computational feasibility analysis is given at first, which is shown in Lemma 2.

**Lemma 2**: The total time complexity of Alg.4 is *O*(*T*_*h*_ + |*E*|/*T*_*h*_).

**Proof**: Since event location algorithm and sensor deployment algorithm are both performed once during initialization, then we only compute the time complexity of data gathering algorithm. For Alg.4. In order to construct the gathering tree, the idea of parallel computing are utilized. Each sensor need to be found once, then the total time complexity is *O*(*T*_*h*_), where *T*_*h*_ presents the height of the tree. On the other hand, each sensor’s neighbourhood need to be scanned, the time complexity is *O*(|*E*|/*T*_*h*_). Then the total time complexity of Alg.4 is *O*(*T*_*h*_ + |*E*|/*T*_*h*_). That is to say the algorithm computation time complexity is feasibility. While other algorithms [8, 9] did not consider the parallel computing, so its total time complexity is *O*(|*V*| + |*E*|), which is higher than ours.

**Fig 12:**
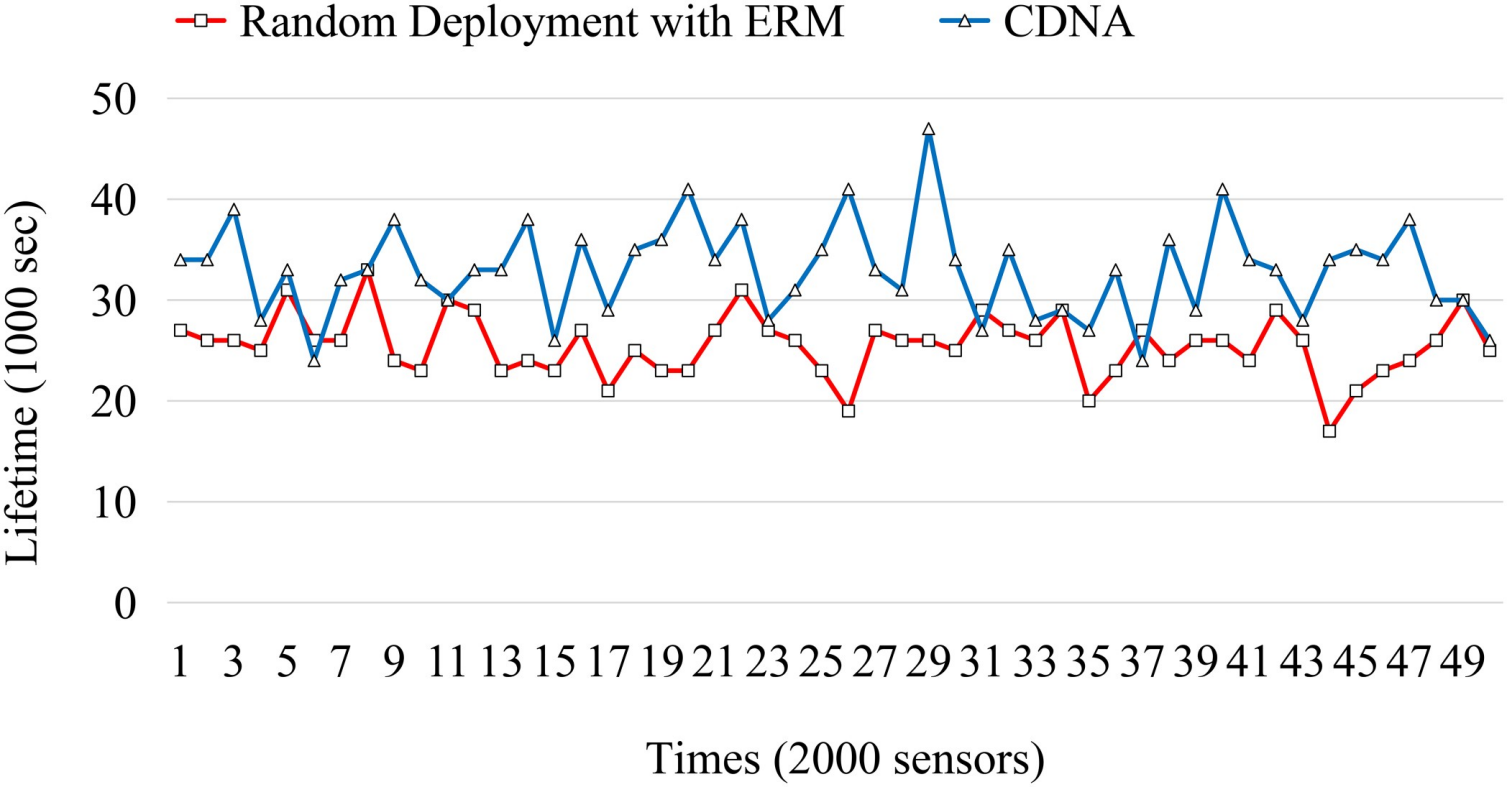
Network lifetime compare for 2000 sensors.

**Fig 13:**
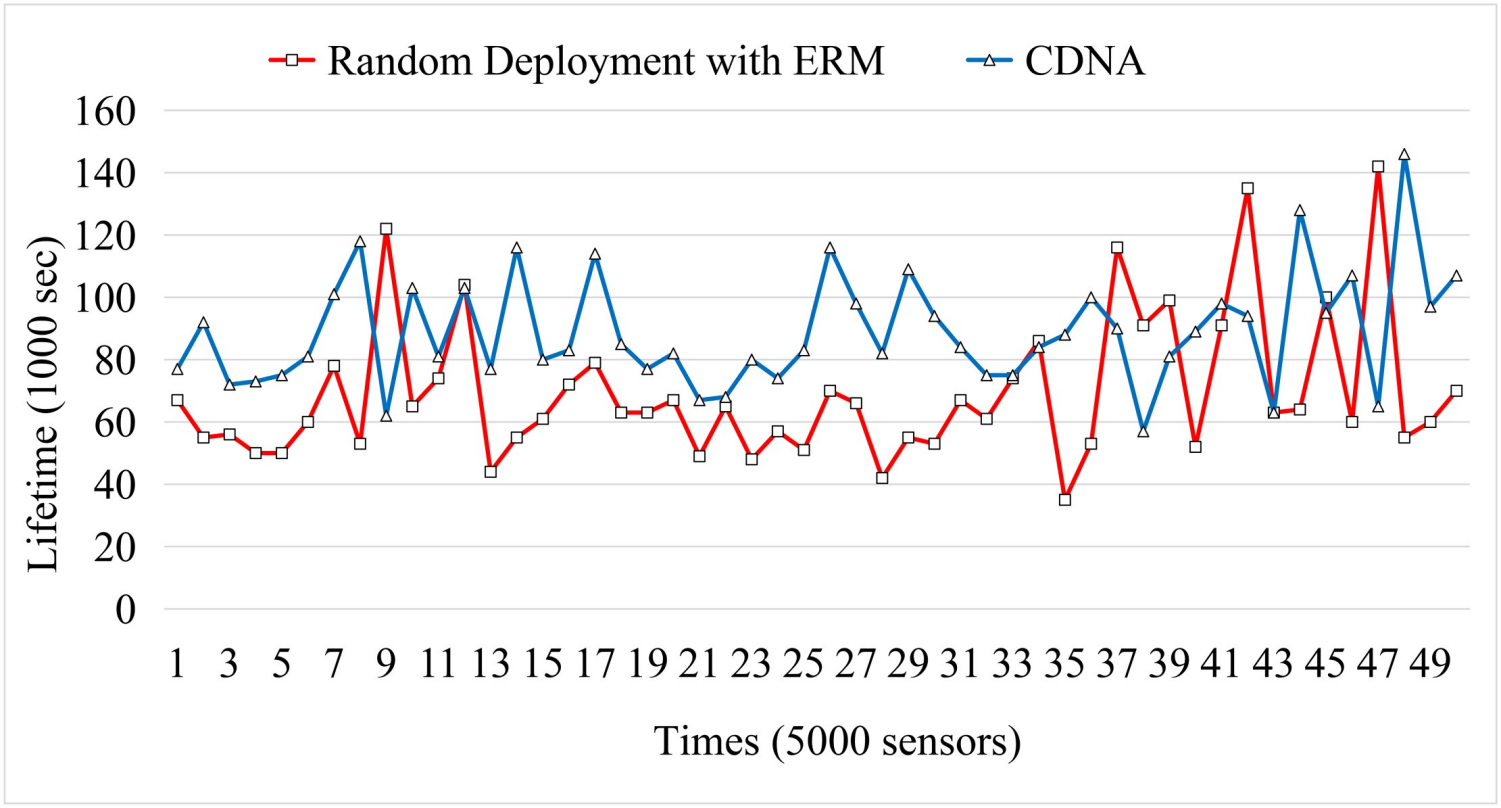
Network lifetime compare for 5000 sensors.

**Fig 14:**
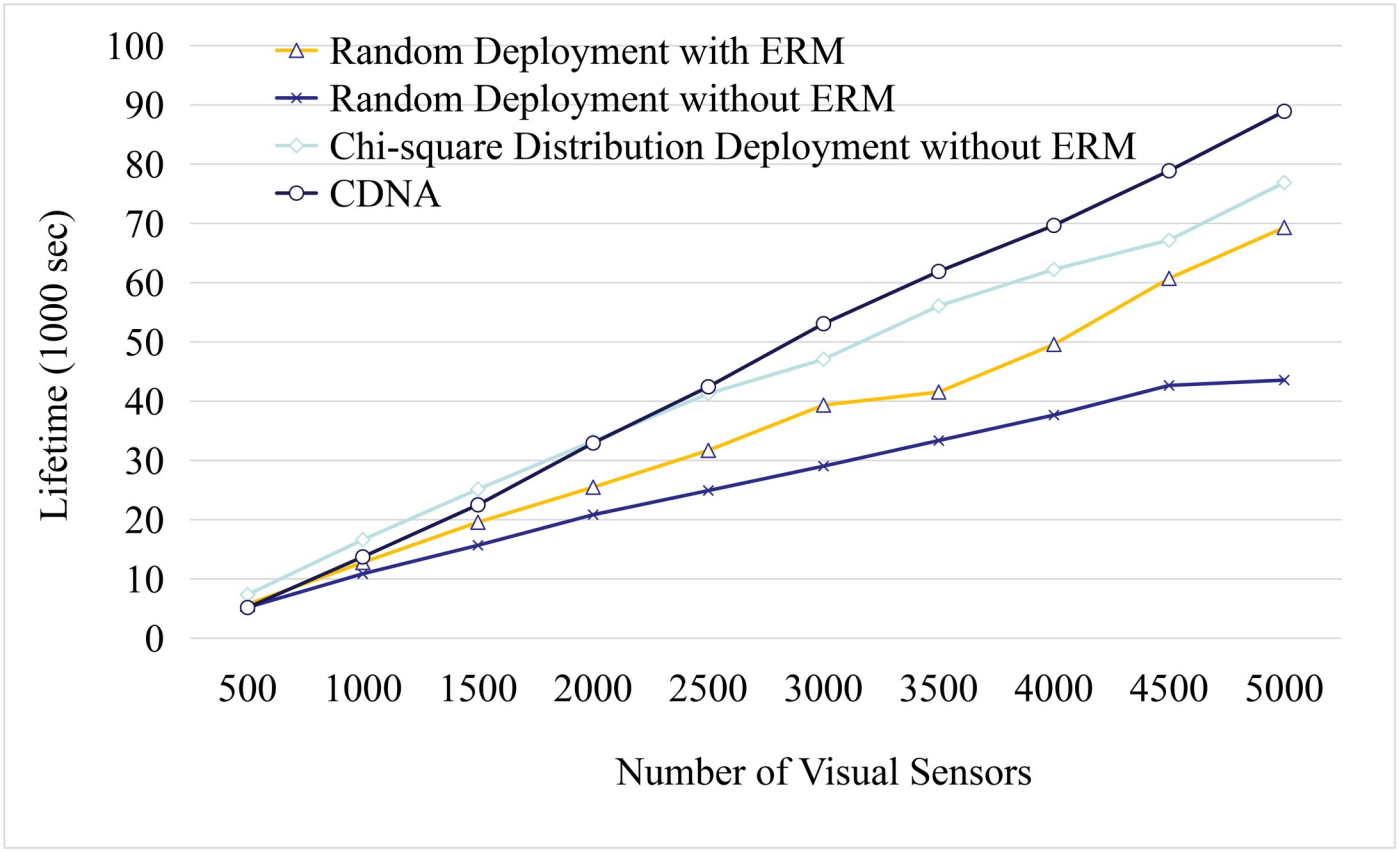
Network lifetime compare.

**Fig 15:**
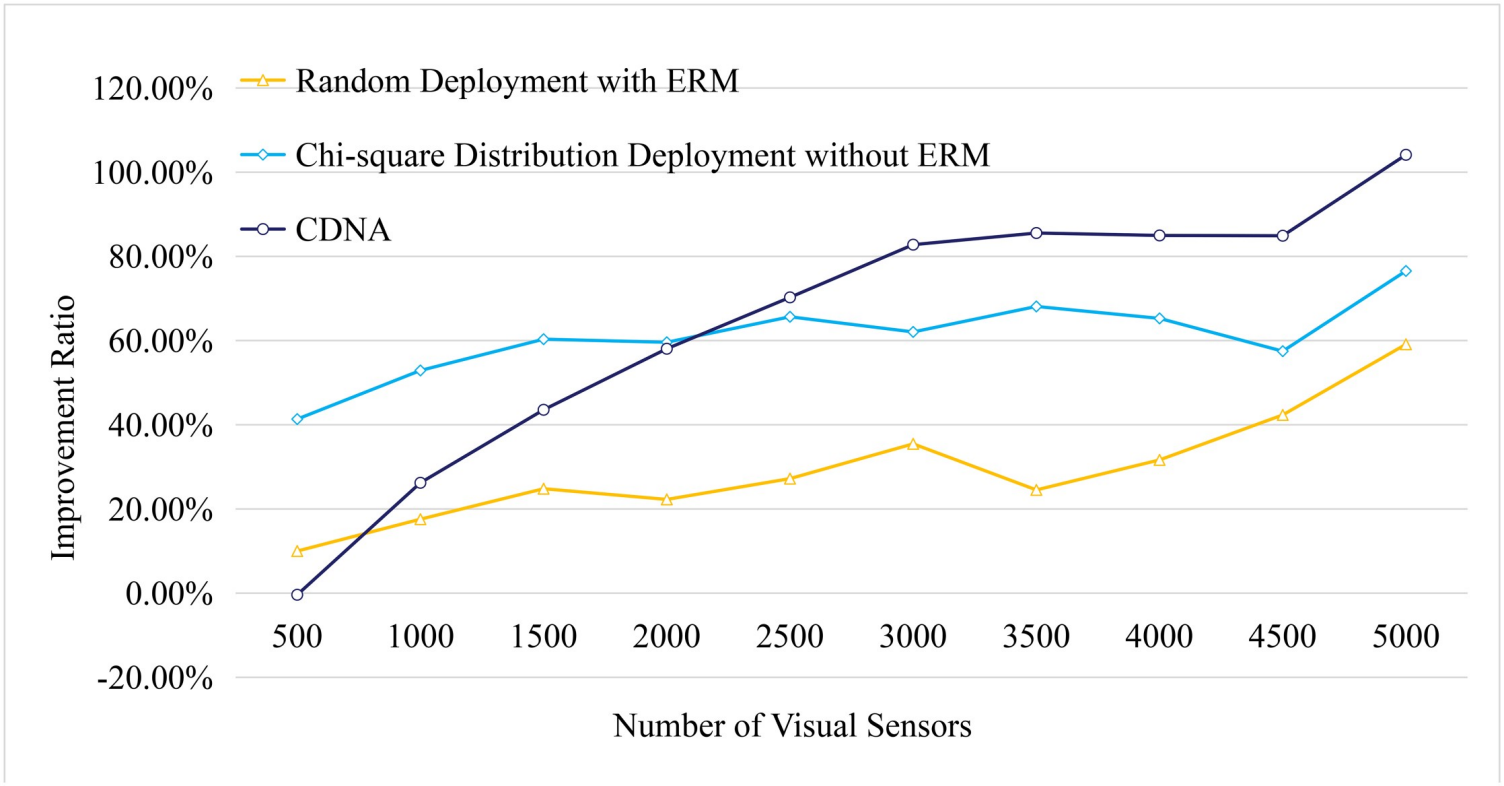
Lifetime comparison.

Next, the number of visual sensors deployed in the space is incrementally increased from 500 to 5000 with the step size of 500 sensors. For each sensor, 10 detectable events are designed in each run. The experimental results are obtained by an average of 50 independent runs. The lifetime compare between the random deployment and the CDNA are shown in Figs 12–15.

The simulation results obtained by the LVSN with 2000 sensors is shown in Fig 13. After removing the top and the worst 10% results in Fig 12, the lifetime of CDNA is extended 28.43% than the random deployment with ERM. As mentioned in the above sections, the *χ*^2^-distribution deployment would assign more sensors to distribute around the sink node compared to the outer regions. These redundant sensors provide the backup solutions for the route construction and eventually prolong the lifetime of the whole network.

The simulation results obtained by the LVSN with 5000 sensors is shown in Fig 13. After removing the top and the worst 10% results in Fig 13, the lifetime of CDNA is extended 31.07% than the random deployment with ERM. Based on the simulation results presented in Figs 12 and 13, the network lifetime is extended longer when the density of the sensors gets higher. That is to say, our proposed CDNA is suitable for large-scale sensor network environments.

The relationship between the total lifetime against the number of sensors is given in Fig 14. Please note that the unit of the vertical axis is thousand-second.

By increasing the total sensors included in the system, the lifetime of the whole network is increased because the number of redundant routes is also increased. Starting with 500 sensors, the lifetime of *χ*^2^-distribution deployment without ERM can prolonged approximately 10% comparing to the random deployment with ERM methods. The improvement gets higher when the included number of sensors increases. When the number of sensors increased to 5000, our CDNA has doubled the network lifetime comparing to the random deployment without ERM. The curves of the *χ*^2^-distribution deployment and the CDNA cross when the number of sensors increases to 2000. It is because the cost of moving sensors is increased and has more impact on energy consumption.

To compare the improvement of the lifetime with different methods, the static random deployment without ERM is used as the baseline. The compare among the mobile random deployment with ERM, the Chi-square distribution deployment without ERM, and the CDNA is given in Fig 15.

As shown in Fig 15, increasing the number of sensors has a positive contribution to prolong the lifetime of the network. When the number increases to more than 3000, the trend of the improvement starts to become stable. In general, the CDNA outperforms the others while Chi-square distribution deployment without ERM and the random deployment with ERM are ranked at the second and the third, respectively.

## Conclusion

The fundamental function of the LVSN is to monitor the specified events and to transmit the detected information back to the sink for achieving the data aggregation purpose. Sensors around the sink generally run out of battery sooner than others because they hold a heavy load on forwarding data from the outside sensors. When sensors in a region are all out of energy, the energy hole problem occurs in that area. Sensor deployment is one of the most crucial issues to solve the energy hole problem. In order to locate the events accurately and prolong the network lifetime, a Comprehensive Visual Data Gathering Network Architecture (CDNA) is proposed in this paper. Three main problems are considered comprehensively in the architecture, which is the first comparatively integrated architecture for LVSNs. 1) A novel *α*-hull based event location algorithm (*α*-ELA), which is oriented from the geometric model of *α*-hull, is designed for accurately and efficiently detect the location of the event. 2) The Chi-Square distribution Event-driven Gradient Deployment algorithm (*χ*^2^-DEGD) is proposed to reduce the unbalanced energy consumption problem to a comparatively low level. 3) An Efficient energy hole Repairing Method (ERM) with two-step processes is designed. Firstly, an Efficient Data Gathering Tree (EDGT) algorithm is proposed for forwarding the visual data, which is the basic premise of the data aggregation. Secondly, a Movement Algorithm (MA) is designed for solving the energy hole problem by moving the redundant sensors to the energy holes areas.

Moreover, the approximation algorithms and the comprehensive theoretical analysis of the approximation factors are presented. The simulation results indicate that our proposed algorithms outperform the existing state-of-the-art approaches significantly. In future work, we intend to focus on when and where the data collection process is needed to eliminate the energy wasted on the event monitoring process. In addition, investigating the Fault-Tolerance virtual backbone for repairing the virtual backbone breakdown caused by the malfunction nodes is a possible way to combine with the proposed CDNA in our future work.
